# Identification and Characterization of a Non-muscular Myostatin in the Nile Tilapia

**DOI:** 10.3389/fendo.2020.00094

**Published:** 2020-02-28

**Authors:** Adi Segev-Hadar, Gertrude Alupo, Kfir Tal, Tali Nitzan, Jakob Biran

**Affiliations:** ^1^Department of Poultry and Aquaculture, Institute of Animal Science, Agricultural Research Organization, Rishon LeTsiyon, Israel; ^2^Department of Animal Sciences, Robert H. Smith Faculty of Agriculture, Food and Environment, The Hebrew University of Jerusalem, Rehovot, Israel

**Keywords:** homeostasis, myostatin (MSTN), nile tilapia (*Oreochromis nilocticus*), environmental challenges, gene duplication

## Abstract

The growth and differentiation factor Myostatin (MSTN, also known as GDF8) negatively regulates skeletal muscle development and growth in vertebrates. Most fish genomes contain two or more *mstn* genes, which are expressed in muscle and other tissues. Yet, in the genome of Nile tilapia (*Oreochromis niloticus*), which is one of the world's most important aquaculture fish species, only one *mstn* gene has previously been identified. Here, we identify a second *mstn* gene in Nile tilapia. We show that it clusters phylogenetically with other piscine *mstn2* genes and that it shares chromosomal synteny with the human and zebrafish orthologs. We further show that *mstn2* is not expressed in red or white muscles of Nile tilapia, but rather that its main site of expression is the brain. To determine which physiological functions are correlated with *mstn* expression, adult Nile tilapia were exposed to various environmental conditions and their effect on *mstn1* and *mstn2* expression in the brain and muscles was measured using real-time PCR. We found that the centrally- and muscle-expressed *mstn* genes differ in their responsiveness to diverse challenges, suggesting differential gene- and tissue-specific regulation of their expression. Metabolic and stress marker analyses showed that the altered *mstn* expression is not regulated by classical stress response. Taken together, our findings expand the understanding of the MSTN system in Nile tilapia and provide evolutionary insight into its function.

## Introduction

Myostatin [MSTN, also known as growth and differentiation factor 8 (GDF8)] is a growth and differentiation factor of the TGF-β superfamily that inhibits skeletal muscle development and growth (1). MSTN is synthesized as a precursor protein, which gives rise to latency-associated peptide and mature MSTN peptide (2). The mammalian *Mstn* gene is mainly expressed in myogenic precursor cells of the myotome during somitogenesis and in muscle tissues of adult animals (1, 3–5). MSTN can act as a paracrine, autocrine, or endocrine substance (6). The *mstn* gene is highly conserved across vertebrate species, supporting its important function in regulating muscular development and growth (1, 7–11).

While mammalian genomes contain only one *Mstn* gene, most ray-finned fish possess at least two *mstn* paralogs (i.e., *mstn1* and *mstn2*) and, in some species, up to four *mstn* genes can be identified (12, 13). Piscine *mstn*s are differentially expressed in different muscle types and, unlike their mammalian orthologs, they are also expressed in the brain and other peripheral tissues (14–16). This tissue distribution suggests that *mstn*s may also be involved in processes such as muscle regeneration, growth, and development of neurons in the brain, osmoregulation, homeostatic tissue growth, and reproduction (14, 15, 17–19). Nevertheless, *mstn* gene knockout in several fish species resulted in increased muscle mass, thus showing the evolutionarily conserved function of *mstn* as a key regulator of muscle growth (20–23).

*mstn* expression is differentially regulated according to species, phase of growth, tissue, nutritional state, stress level, temperature, and activity level (24–27). For example, overcrowding reduced *mstn* mRNA expression in zebrafish (*Danio rerio*) (28), whereas exposure of juvenile channel catfish (*Ictalurus punctatus*) to cold temperature for 28 days increased *mstn* mRNA expression in muscle tissues (24). *mstn* expression is increased in muscles of juvenile European sea bass (*Dicentrarchus labrax*) following prolonged fasting and returns to normal levels after refeeding (29). Thirty days fasting of Asian sea bass (*Lates calcarifer*) fry led to increased expression of *mstn1* in the muscle and liver and decreased expression in the gills and brain. However, *mstn2* expression increased in the gills and liver and remained constant in muscle and brain after long-term fasting (17). Five weeks fasting of juvenile armorhead catfish (*Cranoglanis bouderius*) led to a gradual decrease in *mstn* expression in muscle, brain and liver, which returned to baseline levels after 2 weeks of refeeding. *mstnb* expression increased in the initial fasting period but decreased later on through the prolonged fast (30). In Mozambique tilapia (*Oreochromis mossambicus*) larvae, starvation reduced *mstn1* mRNA levels, accompanied by cortisol elevation. However, fasting of adult male Mozambique tilapia and rainbow trout (*Oncorhynchus mykiss*) had no significant effect on *mstn* expression in skeletal muscles (27, 31). This variability in piscine *mstn* responsiveness emphasizes the need to characterize the piscine MSTN system also in non-muscular tissues under various environmental conditions.

Nile tilapia (*Oreochromis niloticus*) is one of the most widely cultured fish species in extensive and highly intensive aquaculture systems and its genome has been fully sequenced (32–34). It was previously demonstrated that starvation affects *mstn* expression in white muscles of juvenile Nile tilapia (25). However, thus far only one tilapia *mstn* gene has been identified. Here, we report the identification of a non-muscular *mstn2* gene in Nile tilapia and show that it shares phylogeny and chromosomal synteny with *Mstn* genes from invertebrates to mammals. Furthermore, we show that tilapia *mstn1* and *mstn2* differ in their responsiveness to environmental challenges in a tissue-specific manner. Lastly, we show that these effects are mediated by the homeostatic response of the animal to the changing environment and not due to stress response.

## Materials and Methods

### Animals and Treatments

The experiments were approved by the Agricultural Research Organization Committee for Ethics in Using Experimental Animals (approval number: 775/18 IL). Adult Nile tilapia (59.36 ± 2.1 g) were raised in cylindrical 250-liter tanks (*n* = 7–8 fish/treatment) for 5 weeks. Temperature was maintained at 24–26°C. Ammonia and nitrite levels were monitored. Fish were fed twice daily *ad libitum* with commercial tilapia feeds (Zemach Feed Mills™, Israel). Fish were acclimated to the experimental tanks for 1–2 weeks and then subjected for 3 weeks to one of the following treatments: 1. net chasing (10 min/twice daily); 2. 50% seawater (20 ppt; salinity was increased by 0.5% every 48 h during the first 7 days), 3. no feeding; 4. increased water temperature (34°C). Control group underwent no treatment. Each experiment was performed twice in succession and accumulated data from both procedures are presented (*n* = 9–15 fish/treatment). Tissue distribution analysis of *mstn1* and *mstn2* expression was performed using *n* = 3 fish/group/tissue with adult males displaying running milt and weighing 81.87 ± 6.6 g, adult females with fully developed ovaries and weighing 27.13 ± 7.2 g, juvenile males weighing 10.56 ± 1.8 g and juvenile females weighing 16.97 ± 4.4 g.

### Identification of *mstn* Genes in Nile Tilapia

Database sequence searches for *mstn* genes in Nile tilapia were performed using the Basic Local Alignment Search tool (BLAST) package (NCBI, https://blast.ncbi.nlm.nih.gov/Blast.cgi) (35). Genomic synteny of *mstn* was manually analyzed using the UCSC genome browser (https://genome.ucsc.edu/) (36). Nucleotide sequences of the open reading frame of *mstn* genes from various organisms were aligned by MUSCLE and phylogenetic analysis was performed using MEGA version 7 (37).

### Cloning

Nile tilapia total RNA was extracted from brain and muscle tissues using Trizol reagent (Life Technologies Corporation, Carlsbad, USA) according to the manufacturer's protocol and treated with Invitrogen TURBO DNA-free™ kit (Thermo Fisher Scientific, Vilnius, Lithuania) according to the manufacturer's protocol. cDNA was reverse-transcribed from 1 μg total RNA using High Capacity cDNA Reverse Transcription kit (Thermo Fisher Scientific, Vilnius, Lithuania). Specific primer pairs (Table 1) were used to amplify the full tilapia (ti) *mstn2* open reading frame and part of the *timstn1* reading frame. PCR products, amplified with DreamTaq Green PCR Master Mix (Thermo Fisher Scientific), were analyzed on 1% agarose (LifeGene, Modi'in, Israel) containing Redsafe™ stain (Intron Biotechnology, Korea) in 1× TAE (Tris-acetate acid-EDTA) buffer (Biological industries, Kibbutz Beit-Haemek, Israel). PCR products of the predicted amplicon size were extracted from the gel, cloned into pGEM-T easy vector (Promega, Wisconsin, U.S.A.) and sequenced using T7 and SP6 primers at Hy Laboratories Ltd. (Rehovot, Israel).

**Table 1:** Primers used for cloning and real-time PCR (RT).

**Primer**	**Position**	**5^**′**^ to 3^**′**^ sequence**	**Efficiency (%)**	**R^**2**^**	**Application**
tiMSTN1_RT_744F	744	GGGTCTGCAACCGTTCAT	103.456	0.993	RT & cloning
tiMSTN1_RT_863R	863	CAAAGTCCTCGAAGTCCACAG			RT
tiMSTN1_1408R	1,408	TCTATTGCACCGTGTTCTGC			Cloning
tiMSTN2_cloning_1F	1	GCGTCACTGCGCTCACTT			Cloning
tiMSTN2_cloning_1152R	1,152	TAGACATTTCATCCTCAAGGATGC			Cloning
tiMSTN2_RT_234F	234	CAACATCAGCCGCGATATGA			Cloning
tiMSTN2_RT_362R	362	CGATTGGATTGTGCGTTGTTG			Cloning
tiMSTN2_RT_526F	526	GTTCGCTCCCTGAAGATTGA	92.317	0.994	RT
tiMSTN2_RT_640R	640	TTCTATGCCGTAGTGGGTTTC			RT
tiEF1α_F640	640	GGAGACCAGTGACAAGATGAG	97.023	0.987	RT
tiEF1α_R798	798	GTTCCGATACCGCCAATCT			RT
ti18S_897R	897	CGACCATAAACGATGCCAACTAG	98.427	0.999	RT
ti18S_660F	660	GCACCACCACCCACAGAATC			RT

### Tissue and Blood Sample Collection

Blood samples were collected from the lateral vein using 1 mL heparinized syringes (200 IU/mL) attached to 25G needles. Plasma was separated from blood cells and platelets by centrifugation at 4°C/3.2 g for 20 min. Fish were subsequently harvested by decapitation and dissected to collect red muscle, white muscle, forebrain, midbrain, and hindbrain. As in other fish, hypothalamic nuclei of Nile tilapia are localized in the diencephalic compartment (38). Therefore, to analyze central *mstn* expression, the midbrain section containing the diencephalon and optic tectum was dissected from the hindbrain compartments (cerebellum and brain stem) and from the telencephalon and olfactory bulbs. Each brain region was placed in a separate tube. Tissue samples were snap-frozen in liquid nitrogen and stored at −80°C until further analysis. Plasma was stored at −20°C until analysis for triglycerides, total protein and cortisol was performed.

### RNA Extraction and cDNA Synthesis

Tilapia total RNA was extracted using Trizol reagent (Life Technologies Corporation, Carlsbad, USA), according to the manufacturer's protocol. RNA quantity and purity were measured using a microplate spectrophotometer Epoch™ (BioTek instruments Inc. Winooski, USA). RNA integrity was assessed by running 1–1.5 μg of total RNA on 1% agarose (LifeGene, Modi'in, Israel) containing Redsafe™ stain (Intron Biotechnology, Korea) in 1× TAE (Tris-acetate acid-EDTA) buffer (Biological Industries, Kibbutz Beit-Haemek, Israel). Possible genomic DNA contamination was eliminated by treatment with Invitrogen TURBO DNA-free™ kit (Thermo Fisher Scientific, Vilnius, Lithuania) according to the manufacturer's protocol. DNase-free total RNA (0.5 μg) was reverse-transcribed using High Capacity cDNA Reverse Transcription kit (Thermo Fisher Scientific, Vilnius, Lithuania) according to the manufacturer's protocol. cDNA was stored at −20°C until quantitation by real-time PCR.

### Real-Time PCR Analysis

Expression levels of *timstn1* in red and white muscle and *timstn1* and *timstn2* in the brain were analyzed by quantitative PCR using a StepOnePlus™ Real-Time PCR System (Applied Biosystems, Inc. Foster City, CA, USA). Elongation factor 1 alpha (*ef1*α) and 18S served as reference genes (39). Each reaction consisted of 5 μL SYBR® green dye (Thermo Fisher Scientific, Vilnius, Lithuania), 0.75 μL of 3 μM forward and reverse primers of either *timstn1, timstn2, 18s*, or *ef1*α (Table 1), 0.5 μL of ultra-pure water (UPW) and 3 μL of cDNA template (diluted 1:20 in UPW for brain and white muscle and 1:10 for red muscle). Analysis was performed in duplicates. Controls without the cDNA were used to test for non-specific amplification. Specificity of the primers was validated by Sanger sequencing and melt curve analysis was used to confirm amplification of a single product. Amplification was performed under the following conditions; 95.0°C for 20 s, 40 cycles at 95.0°C for 3 s, and 60.0°C for 30 s, followed by one cycle at 95.0°C for 15 s and 60.0°C for 1 min, 95.0°C for 15 s for the generation of the melting curve. Fluorescence signals of the target, reference genes and control group were analyzed using StepOne software Version 2.3. Tissue distribution analysis had no clear baseline; therefore, relative quantification in various tissues was performed using 2^−ΔC'T^ method (40). Relative quantification of within-tissue expression was determined using the 2^−ΔΔCT^ method (41).

### Quantification of Metabolites and Cortisol in Plasma

Plasma metabolites (triglycerides and total protein) were quantified by a photometric method using Cobas™ C111 Chemistry Analyzer (Roche Diagnostics International Ltd. Rotkruez, Switzerland). The system was calibrated using tetramethylammonium chloride (C.f.a.s Calibrator; Roche diagnostics GmbH, Mannheim Germany). Analytic controls (PreciControl ClinChem Multi 1 and 2; Roche diagnostics GmbH, Mannheim Germany) consisting of lyophilized human sera were used for quality control. Triglycerides were quantified using the TRIGL kit (Roche diagnostics GmbH, Mannheim, Germany) and total protein concentration was measured using the TP2 Kit (Roche diagnostics GmbH) according to the manufacturer's protocols. Steroid extraction for cortisol analysis was performed according to Aizen et al. (42) and cortisol concentrations were measured using a cortisol-specific ELISA according to the protocol published by Yeh et al. (43).

### Statistical Analyses

Statistical analyses were performed using GraphPad Prism 7.01 software (GraphPad, San Diego, USA). Data are presented as mean ± SD. Significance of differential gene expression and metabolic parameters was determined by one-way ANOVA followed by Dunnett's multiple comparisons post-test.

## Results

### Identification of a Second *mstn* Gene in the Genome of Nile Tilapia

**Figure 1:**
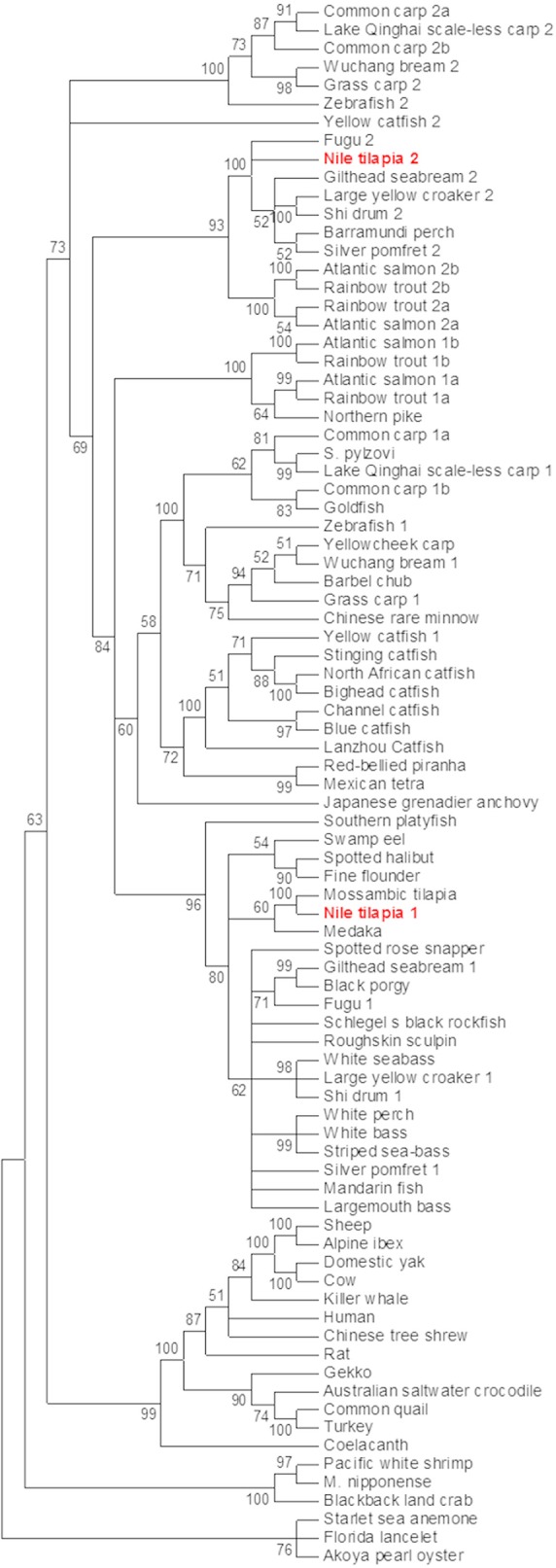
Phylogenetic analysis of *mstn* nucleotide sequences. The *mstn* genes cluster into five main clades including an invertebrate clade, a clade of reptiles and avians, a mammalian clade that also includes the ancient coelacanth fish, a clade of piscine *mstn1* and a clade for piscine *mstn2*. Sequences used in this analysis are: Mozambique tilapia, *Oreochromis mossambicus*, AF197193; Lanzhou catfish, *Silurus lanzhouensis*, KU302769; Nile tilapia 1, *Oreochromis niloticus*, XM_003458832; Nile tilapia 2, *Oreochromis niloticus*, MN708486; Silver pomfret 1, *Pampus argenteus*, KM259900; Lake Qinghai scale-less carp 1, *Gymnocypris przewalskii*, KJ607138; Japanese grenadier anchovy, *Coilia nasus*, KF638401; *Macrobrachium nipponense*, KF530847; Roughskin sculpin, *Trachidermus fasciatus*, GU198192; goldfish, *Carassius auratus*, KC851952; Chinese rare minnow, *Gobiocypris rarus*, FJ482232; starlet sea anemone, *Nematostella vectensis*, AGL96595; common quail, *Coturnix coturnix*, AF407340; spotted rose snapper, *Lutjanus guttatus*, JX987064; spotted halibut, *Verasper variegatus*, JN226745; Barbel chub, *Squaliobarbus curriculus*, JN230816; Barramundi perch, *Lates calcarifer* HQ731440; cow, *Bos taurus*, GQ184147; yellowcheek carp, *Elopichthys bambusa*, HM461971; white perch, *Morone Americana*, AF290911; striped sea-bass, *Morone saxatilis*, AF290910; gilthead seabream 1, *Sparus aurata*, AF258448; white bass, *Morone chrysops*, AF197194; turkey, *Meleagris gallopavo*, AF019625; human, *Homo sapiens*, AF104922; Norway rat, *Rattus norvegicus*, AF019624; sheep, *Ovis aries*, AF019622; common carp 1a, *Cyprinus carpio*, GU014395; common carp 1b, *Cyprinus carpio*, GU014396; common carp 2a, *Cyprinus carpio*, GU014397; common carp 2b, *Cyprinus carpio*, GU0143958; yellow catfish 1, *Tachysurus fulvidraco*, DQ767966; Alpine ibex, *Capra ibex*, AY629305; Schlegel's black rockfish, *Sebastes schlegelii*, DQ423474; blackback land crab, *Gecarcinus lateralis*, EU432218; black porgy, *Acanthopagrus schlegelii*, DQ303480; white seabass, *Atractoscion nobilis*, AY966401; stinging catfish, *Heteropneustes fossilis*, HQ003245; North African catfish, *Clarias gariepinus*, KJ372760; swamp eel, *Monopterus albus*, KM103284; Akoya pearl oyster, *Pinctada martensii*, KJ579132; *Schizopygopsis pylzovi*, JX088635; bighead catfish, *Clarias microcephalus*, JX456396; Mandarin fish, *Siniperca chuatsi*, JF896453; blue catfish, *Ictalurus furcatus*, AY540992; largemouth bass, *Micropterus salmoides*, DQ666527; Florida lancelet, *Branchiostoma floridae*, XM_002599415; domestic yak, *Bos grunniens*, EU926669; zebrafish a, *Danio rerio*, NM_001004122; zebrafish b, *Danio rerio* NM_131019; fugu 2, *Takifugu rubripes*, NM_001032672; Lake Qinghai scale-less carp 2, *Gymnocypris przewalskii*, KP277103; grass carp 2, *Ctenopharyngodon idella*, KM874827; grass carp 1, *Ctenopharyngodon idella*, KM874826; Japanese medaka, *Oryzias latipes*, NM_001201499; yellow catfish 2, *Tachysurus fulvidraco* KF537384; shi drum 2, *Umbrina cirrosa*, JX002683; Wuchang bream 2, *Megalobrama amblycephala*, JQ065337; Wuchang bream 1, *Megalobrama amblycephala*, JQ065336; fugu 1, *Takifugu rubripes*, AY445322; fine flounder 1, *Paralichthys adspersus*, EU443627; Australian saltwater crocodile, *Crocodylus porosus*, XM_019554060; large yellow croaker 1, *Larimichthys crocea*, AY842933; red-bellied piranha, *Pygocentrus nattereri*, XM_017713514; gecko, *Gekko japonicas*, XM_015405581; Mexican tetra, *Astyanax mexicanus*, XM_007253246; coelacanth, *Latimeria chalumnae*, XM_005996542; Chinese tree shrew, *Tupaia chinensis*, XM_006147825; channel catfish, *Ictalurus punctatus*, XM_017469117; southern platyfish, *Xiphophorus maculatus* XM_014468464; gilthead seabream 2, *Sparus aurata*, AY046314; large yellow croaker 2, *Larimichthys crocea*, JF304776; Pacific white shrimp, *Litopenaeus vannamei*, JQ045427; northern pike, *Esox Lucius*, XM_010879812; killer whale, *Orcinus orca*, XM_004276934; shi drum 1, *Umbrina cirrosa*, AF316881; silver pomfret 2, *Pampus argenteus*, KT726407; rainbow trout 1a, *Oncorhynchus mykiss*,NM_001124282; rainbow trout 1b, *Oncorhynchus mykiss*,NM_001124283; rainbow trout 2a, *Oncorhynchus mykiss*,DQ417326; rainbow trout 2b (pseudogene), *Oncorhynchus mykiss*, DQ417327; Atlantic salmon 1a, *Salmo salar*, NM_001123634; Atlantic salmon 1b, *Salmo salar*, NM_001123549; Atlantic salmon 2a, *Salmo salar*, JN990763; Atlantic salmon 2b (pseudogene), *Salmo salar*, JN990773. Phylogenetic analysis was performed using MEGA7 software (37). The evolutionary history was inferred by using the maximum likelihood method based on the Tamura-Nei model (44). The percentage of replicate trees in which the associated taxa clustered together in the bootstrap test (500 replicates) are shown next to the branches (45). Initial tree(s) for the heuristic search were obtained automatically by applying Neighbor-Join and BioNJ algorithms to a matrix of pairwise distances estimated using the maximum composite likelihood (MCL) approach, and then selecting the topology with superior log likelihood value. The analysis involved 85 nucleotide sequences.

Nile tilapia *mstn* genes were sought using the previously identified Mozambique tilapia *mstn* mRNA sequence (AF197193) (15). A standard BLASTn search against the NR database of Nile tilapia yielded two predicted *mstn* mRNAs (XM_003458832 and XM_003446535), with the first hit matching the previously cloned *mstn* gene of Nile tilapia (KT987208). A wide-range phylogenetic analysis of the *Mstn* open reading frame (ORF) from invertebrates and lower vertebrates to mammals showed that the analyzed *mstn* sequences cluster into five main clades: 1. invertebrates *mstn*; 2. reptile and avian *mstn*; 3. mammalian *Mstn*; 4 piscine *mstn1*; 5. piscine *mstn2*. The previously identified tilapia *mstn* (*timstn1*) clustered with other piscine *mstn1* ORFs, whereas the newly identified *timstn* clustered with *mstn2* ORFs of various fish (Figure 1) and was therefore designated *timstn2*.

**Figure 2:**
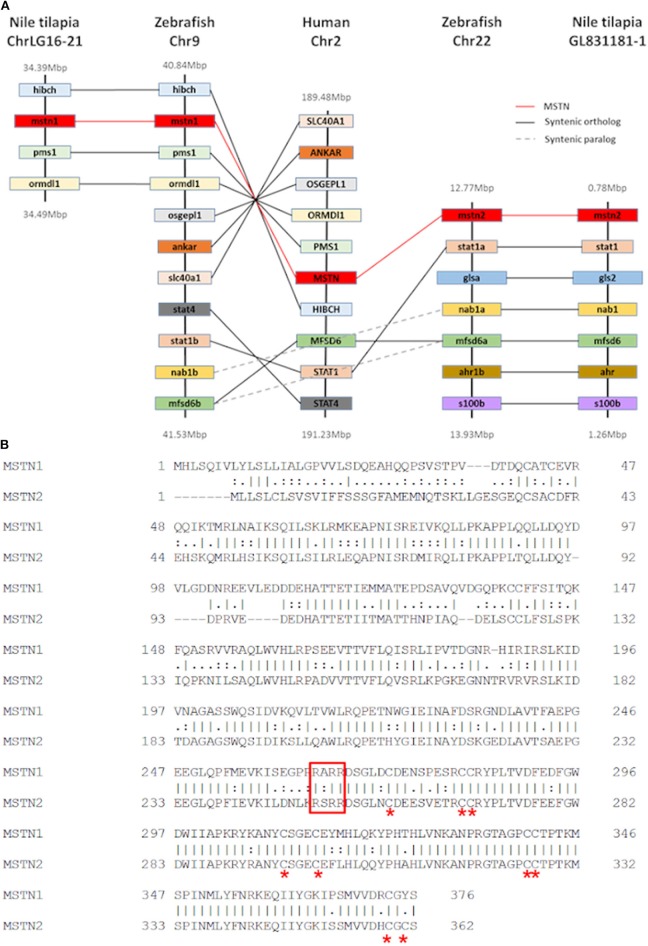
Conservation of tilapia *mstn2*. **(A)** Chromosomal synteny of Nile tilapia *mstn1* and *mstn2*. Genes adjacent to *mstn* in the Nile tilapia, zebrafish and human genomes were manually identified using both USCS and ensembl genome browsers (https://genome.ucsc.edu/ and https://www.ensembl.org/index.html, respectively). The genes are named according to their annotation in the human genome. While Nab1 and MFSD6a genes were found to be syntenic in both piscine *mstn* genomic regions, most of the human *MSTN* neighboring genes were syntenic only to *mstn1* or *mstn2*. **(B)** Pairwise alignment of tilapia Mstn1 and Mstn2 amino acid sequences illustrates the high similarity of the tilapia Mstn proteins. Red asterisk indicates conserved cysteine residues, which are important for Mstn peptide activity. The conserved proteolytic RXRR motif is indicated by a red rectangle.

Although our analysis may have been biased by the increased number of piscine species, the results strongly support an early evolutionary event of *mstn* gene duplication in fish genomes. To further test this hypothesis, we analyzed the syntenic conservation between the *timstn*s neighboring genes and zebrafish *mstn*s genomic regions using the human *MSTN* gene as a reference. This analysis demonstrated high syntenic conservation between the chromosomal regions of human *MSTN* and zebrafish *mstn1* (a.k.a. *mstnb*; NM_131019). Furthermore, syntenic conservation to the chromosomal region of the human *MSTN* gene was also found for both of the *timstn*s and for zebrafish *mstn2* (a.k.a. *mstna*; NM_001004122). The MFSD6 was identified in chromosomal region of the human *MSTN, timstn2*, zebrafish *mstn1*, and *mstn2*, whereas *Nab1* gene was conserved in *timstn2* and both of the zebrafish *mstn* genes (Figure 2A). These findings suggest that the mammalian *Mstn* gene shares a common ancestral gene with the piscine *mstn* genes and that both of the piscine *mstn* genes retained at least part of their chromosomal synteny during speciation events.

While the Nile tilapia *mstn1* gene has already been cloned, the *timstn2* prediction was based on computational annotation. Although partial cloning of *timstn1* was successful, our efforts to clone *timstn2* from cDNA libraries of tilapia muscles failed. Nonetheless, the full ORF of *timstn2* was cloned from brain cDNA library (accession no: MN708486), suggesting a differential role for *timstn2*. At the protein level, tiMstn1 and the newly cloned tiMstn2 shared 69% identity and 82% similarity, with higher homology in the C-termini of the peptide (Figure 2B; Table 2). Homology rate analysis showed that tiMstn peptide sequences share 63–86% homology and 78–92% similarity with MSTN peptides of other vertebrates, whereas tiMstn2 shared 60–76% homology and 74–86% similarity (Table 2). Moreover, both of the translated tiMstns possessed the characteristic MSTN cysteine residues and proteolytic RXRR site (Figure 2B) (16).

**Table 2:** Comparison of the homology of myostatin (MSTN) protein sequences in Nile tilapia with other species.

**Protein**	**Nile tilapia 1**	**Nile tilapia 2**	**Human**	**Mouse**	**Cow**	**Salmon 1a**	**Salmon 1b**	**Salmon 2a**	**Medaka**	**Zebrafish 1(b)**	**Zebrafish 2(a)**
**Nile tilapia 1**		**69**	**66**	**65**	**63**	**83**	**82**	**66**	**86**	**80**	**64**
**Nile tilapia 2**	82		**60**	**63**	**63**	**67**	**66**	**76**	**68**	**71**	**67**
**Human**	80	74		**96**	**94**	**66**	**66**	**59**	**64**	**68**	**64**
**Mouse**	79	76	98		**93**	**65**	**65**	**59**	**63**	**67**	**63**
**Cow**	78	76	96	95		**64**	**64**	**58**	**62**	**66**	**63**
**Salmon 1a**	90	77	78	78	77		**93**	**66**	**83**	**86**	**65**
**Salmon 1b**	91	76	79	79	77	95		**68**	**80**	**85**	**66**
**Salmon 2a**	80	86	74	73	73	77	79		**64**	**66**	**64**
**Medaka**	92	79	77	76	75	88	88	77		**80**	**64**
**Zebrafish 1(b)**	90	82	82	81	76	90	91	78	87		**70**
**Zebrafish 2(a)**	78	80	77	77	80	78	78	80	77	82	

*Table comparing of human, mice, cattle, salmon, Medaka, and zebrafish. Percentage of identity (in bold) and of similarity are shown of protein sequences of Nile tilapia (Oreochromis niloticus) myostatin1 (accession number XP_003458880); Nile tilapia myostatin2 (MN708486); human (Homo sapiens) growth/differentiation factor 8 preproprotein (NP_005250); mouse (Mus musculus) growth/differentiation factor 8 preproprotein (NP_034964); cattle (Bos taurus) growth/differentiation factor 8 precursor (NP_001001525); salmon (Salmo salar) myostatin 1a (ABN72586); salmon myostatin 1b precursor (NP_001117106); salmon myostatin 2a (ABN72587); medaka (Oryzias latipes) growth/differentiation factor 8 precursor (NP_001188428); zebrafish (Danio rerio) myostatin a precursor (NP_001004122); and zebrafish growth/differentiation factor 8 preproprotein (NP_571094). Salmon myostatin 2a is a pseudogene, hence it is not translated into a protein. Regions of local similarity between the protein sequences above were identified using BLAST*.

### Tilapia *mstn* Genes Differ in Their Tissue Distribution

**Figure 3:**
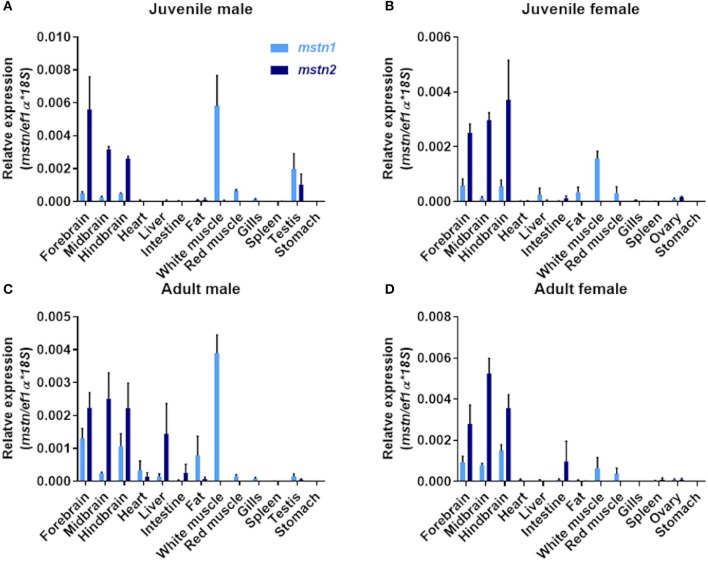
Localization of *timstn1* and *timstn2* mRNA in various tissues of Nile tilapia using real-time PCR. Expression levels of *timstn1* and *timstn2* were determined by real-time PCR in juvenile male **(A)**, juvenile females **(B)**, adult males **(C)**, and adult females **(D)**. The data are presented as mean ± SD.

Piscine *mstn* genes are expressed in multiple tissues (16). Therefore, the identification of *mstn* gene duplication raises the question of whether both tilapia *mstn*s regulate muscle development and growth. To address this point, we performed quantification of *timstn1* and *timstn2* mRNA expression in various tissues of Nile tilapia, using real-time PCR. Because tilapia males exhibit increased growth rate as compared to females and gonadal development was shown to influence muscle growth rate in tilapia (46, 47), we analyzed the expression of *timstn1* and *timstn2* in both sexes before and after sexual maturation. This analysis showed that male tilapia express higher levels of *timstn1* in their white muscle than females before and after sexual maturation (Figure 3). The highest levels of *timstn1* were found in white muscle tissue. Intermediate levels of *timstn1* expression were found in the red muscle and brain of all groups, in the fat of mature males and in the testis of premature males. Low levels of *timstn1* were found in the ovary of mature and premature females, the mature male testis and the liver of all groups. Interestingly, *timstn2* was not expressed in red or white muscles and its highest expression was detected in the brains of all tested groups. Lower levels of *timstn2* expression were detected also in the intestine and gonads before and after sexual maturation (Figure 3).

### Environmental Conditions Influence the Expression of *mstn1* and *mstn2* in the Brain and Muscles of Nile Tilapia

**Figure 4:**
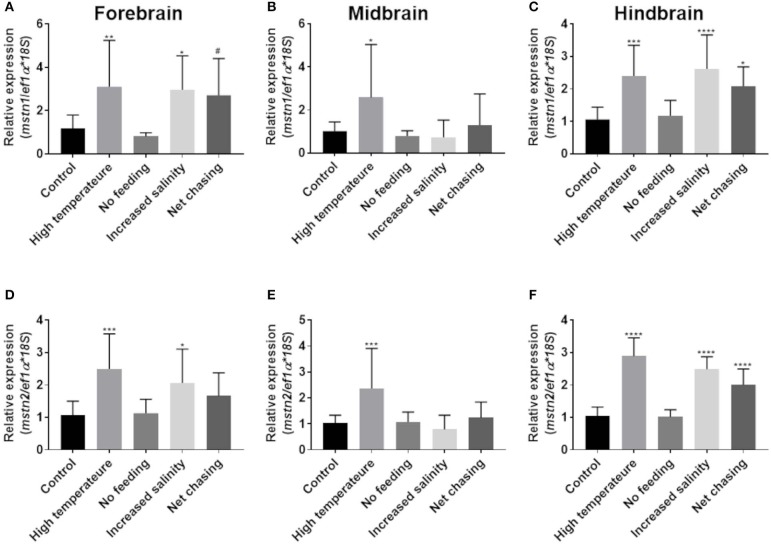
Environmental challenges affect the expression of *timstn1* and *timstn2* in Nile tilapia brain. Expression levels of *timstn1* **(A–C)** and *timstn2* **(D–F)** were determined by real-time PCR. Expression was analyzed in the fish forebrain **(A,D)**, midbrain **(B,E)**, and hindbrain **(C,F)**. The data are presented as mean ± SD. *p < 0.05; **p < 0.01; ***p < 0.001; ****p < 0.0001; ^#^*p* = 0.053.

Fluctuations in environmental conditions are known to induce hypothalamic activity as part of the homeostatic response to the changes (48). In addition, the piscine cerebellum and, to a lesser extent, the telencephalon and diencephalon are major sites of adult brain neurogenesis, and adult brain size can also be affected by environmental conditions (49, 50). To test whether *mstn* is involved in the regulation of these functions, fish were exposed to various changes in conditions (see Materials and Methods) for 3 weeks and analyzed for *timstn* gene expression in the forebrain, midbrain and hindbrain. The results showed that increased water temperature led to increased *timstn1* mRNA expression in all brain regions (Figures 4A–C). Additionally, increased salinity led to increased *timstn1* expression in the forebrain and hindbrain compartments and net chasing treatment led to increased *timstn1* expression in the hindbrain, with a strong trend for increased expression in the forebrain (Figures 4A,C). Similarly, *timstn2* mRNA expression significantly increased in response to high water temperature in all brain regions (Figures 4D–F). Forebrain and hindbrain *timstn2* expression rose in response to increased salinity; however, net chasing elicited *timstn2* expression only in the hindbrain compartment (Figures 4D,F). Interestingly, lack of food for 3 weeks did not affect *timstn1* or *timstn2* mRNA expression in the tilapia brain.

**Figure 5:**
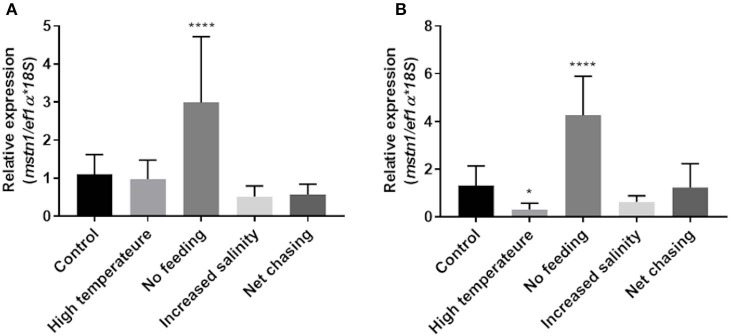
Environmental challenges affect the expression of *timstn1* in Nile tilapia muscle tissues. Expression levels of *timstn1* mRNA in the white muscle **(A)** and red muscle **(B)** were determined by real-time PCR. The data are presented as mean ± SD. *p < 0.05; ****p < 0.0001.

Considering its highly conserved role in regulating muscle growth in various fish, we next examined how environmental challenges affect *mstn* expression in the Nile tilapia muscles. Having found that *timstn2* is not expressed in the fish muscles (Figure 3), only *timstn1* expression was analyzed. In white muscle tissue, *timstn1* expression significantly increased in response to starvation, while other treatments did not affect its expression (Figure 5A). Similar results were seen in red muscle tissue, where only starvation elicited a significant increase in *timstn1* expression. Interestingly, heat treatment led to a significant reduction of *timstn1* expression in the red muscle (Figure 5B).

### The Effect of Environmental Challenges on Protein and Triglyceride Metabolism

**Figure 6:**
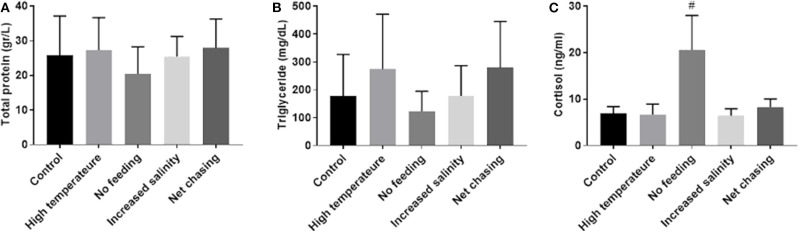
Metabolic parameters in plasma of Nile tilapia remained unchanged in response to various environmental conditions. Total protein **(A)** and triglyceride **(B)** levels were determined by photometric measurement. Plasma cortisol levels **(C)** were analyzed using a specific ELISA. The data are presented as mean ± SD. ^#^*p* = 0.098.

Stress induces a rise in cortisol that, in turn, influences metabolism of carbohydrates, lipids and proteins (51). Moreover, cortisol can influence *mstn* expression in several fish species, including tilapia (31, 52). Therefore, to examine the effect of the above-mentioned experimental challenges on energy metabolism in Nile tilapia, we analyzed total protein and triglyceride levels in the plasma. Our analysis revealed that total protein levels in plasma did not change in response to any of the experimental treatments (Figure 6A). Similarly, none of the experimental treatments affected plasma levels of triglycerides (Figure 6B). These findings were unexpected, as in an acute form, these challenges will induce a homeostatic stress response. We therefore tested cortisol levels in the plasmas of the experimental fish. Results showed a trend for increased cortisol levels in the no-feed treatment group, but no effect for any of the other treatments (Figure 6C).

## Discussion

The majority of fish genomes contain multiple copies of *mstn* that are expressed in various tissues including the muscles, brain, gonads, liver and others. Moreover, within a given species, paralogous *mstn* genes may differ in their tissue distribution (16). This suggests that in addition to their role as key regulators of muscle development and growth, fish *mstn* genes may have pleiotropic roles in the brain and periphery (16). So far, only one *mstn* gene, namely *mstn1*, has been investigated in Nile tilapia (25, 26). Herein, we describe the identification of a second, non-muscular *mstn* gene in the genome of this species. We show that the newly identified *timstn2* clusters phylogenetically with other known piscine *mstn2* genes and that it is mainly expressed in the fish brain, suggesting its involvement in the regulation of central functions. We therefore exposed adult Nile tilapia to diverse environmental conditions and found that the expression of the two *timstn* genes varies with tissue and environmental conditions, supportive of separate regulatory mechanisms for each gene. Importantly, analysis of metabolic markers and cortisol demonstrated that the modified expression of *timstn* is not driven by classical stress response.

Phylogenetic analysis of *mstn* ORFs showed that piscine *mstn* genes form two clusters, one that contains *mstn1* ORFs, including that of Nile tilapia, and another containing *mstn2* ORFs, including the newly identified Nile tilapia *mstn2*. As demonstrated for other fish, the *mstn* gene duplication in tilapia is probably a result of a genome duplication event that occurred in a common ancestor during fish evolution (53). Furthermore, the two *timstn* genes share chromosomal synteny with zebrafish and human *Mstn* genes. At the peptide level, both tilapia Mstns share relatively high homology with MSTNs of other vertebrates.

Although piscine *mstn* genes were suggested to have pleiotropic functions, several works have demonstrated their importance as regulators of muscle mass in fish (17, 20, 54). Fish display significant change in growth rates following sexual maturation, and Nile tilapia males are known to grow faster than females (46, 47). Therefore, we analyzed the tissue distribution of the *mstn* genes in male and female tilapia before and after sexual maturation. Real-time PCR analysis showed that male tilapia expressed more *timstn1* in their muscle than females before and after sexual maturation, suggesting sexual dimorphism in *timstn1* expression. Similarly, female rare minnow (*Gobiocypris rarus*) expressed lower levels of *mstn* than the males in their muscles. Nonetheless, in both sexes *mstn1* expression is highest in white muscles (55). Low to intermediate levels of *timstn1* expression were found in red muscles and brain of all groups, in the fat of mature males, in the liver and in the gonads of both sexes at both developmental stages, suggesting the involvement of *timstn1* in additional metabolic and reproductive functions. In both sexes and maturation stages, the highest expression of *timstn2* was found in the brain. Importantly, *timstn2* was not detected in red or white muscles of all tested groups. Low levels of *timstn2* were also detected in the fish intestine and gonads, both before and after sexual development. A similar pattern was observed in 2-year-old gilthead seabream (*Sparus aurata*), where high *mstn2* expression was evident in the brain with minimal expression in the gonads and no expression at all in the muscle (18). The presence of *mstn2* in the brain and gonads suggests an additional role for MSTN in fish, such as neuronal development or regulation of reproductive functions.

Several studies have demonstrated that exposing fish to environmental challenges, such as food shortage or temperature changes, lead to alterations in *mstn* expression. Yet, the responsiveness of *mstn* seems to vary between species and challenges (16). We therefore tested how various environmental challenges influence *mstn* expression in the fish brain and muscles. Most of the challenges we employed elicit a homeostatic response in the hypothalamus (48). Therefore, we analyzed *mstn* expression separately in the forebrain, midbrain, and hindbrain. Exposing the fish to increased water temperature led to increased expression of *timstn1* and *timstn2* in the brain, indicating the involvement of the Nile tilapia Mstn system in the central response to environmental challenges. Conversely, high temperature treatment did not affect *timstn1* expression in the white muscle and led to a significant decrease of *timstn1* in the red muscle, suggesting that *timstn1* expression is regulated in a tissue-specific manner. Exposing the fish to increased salinity led to increased expression of *timstn1* and *timstn2* in the forebrain and hindbrain, while net chasing elicited a significant increase in *timstn1* and *timstn2* expression only in the hindbrain. The hindbrain compartment comprises the cerebellum and brain stem, whereas the forebrain compartment contains the telencephalon and olfactory bulbs. The cerebellum and, to a lesser extent, the telencephalon are major sites for neurogenesis in the adult fish brain (50). Furthermore, it has been demonstrated that environmental conditions may affect brain size (49). Thus, the increased *mstn* expression in the hindbrain and forebrain suggests the involvement of these genes in the regulation of tilapia neurogenesis. Furthermore, the modified expression of *mstn* in the various brain compartments of Nile tilapia support different functions of the MSTN system according to the site of expression. The midbrain compartment included the fish hypothalamus and optic tectum. Thus, alterations in *mstn* expression in the tilapia midbrain could reflect involvement in the neuroendocrine homeostatic response to the altered environmental conditions. Whether the increased expression of tilapia *mstn*s in the midbrain is neuroendocrine or mitogenesis-related by nature remains to be determined.

In the muscle, MSTN was shown to suppress satellite cell proliferation from fish to mammals (56, 57). Three weeks of starvation led to increased *timstn1* expression in both red and white muscles. Considering the key role of *mstn1* as a suppressor of muscle growth, this increase was probably intended to inhibit muscles growth during energetic shortage. The observed reduction in *timstn1* expression in red muscle may support increased growth and metabolic rate of a poikilothermic animal in a warmer environment. Other treatments did not elicit *timstn1* expression in white or red muscles. European sea bass larvae exposed to heat shock or handling did not display altered *mstn* expression; however, that analysis was performed on whole larvae and under acute challenges (58) and, therefore, it cannot be correlated with our findings. Taken together, these findings suggest that the expression of *timstn*s in the fish brain and muscles is regulated by different mechanisms. These findings are in agreement with other studies in Asian sea bass fry and European sea bass (17, 29).

Environmental stressors stimulate the secretion of stress hormones such as cortisol. Once in circulation, cortisol initiates a secondary response, which involves metabolic changes with aim to restore homeostasis (51). It is not clear how chronic challenges of intensive handling, high salinity, temperature manipulation, and food deprivation affect plasma metabolites such as proteins and triglycerides in Nile tilapia. Skeletal muscle tissue is a major reservoir of body proteins, and muscle mass constitutes a large part of the fish body weight. Therefore, the fish body mass may be influenced by the degree of protein synthesis or degradation (59). Total protein and triglycerides levels in the fish plasma remained unaffected in all treatment groups. As the fish were fed *ad libitum* prior to the experimental starvation, it is possible that the energetic shortage was compensated by utilization of fat storage. This notion is further supported by the observed cortisol levels, which support low activity of the neuroendocrine stress axis. Taken together, these findings indicate that under the applied experimental conditions, alterations in *timstn* expression are not conveyed by stress responses to the challenges. Moreover, these alterations were probably part of the fish habituation to the new environmental conditions.

In summary, in this study we have identified a second *mstn* gene in the Nile tilapia genome and designated it *timstn2*. We have shown that *timstn1* and *timstn2* differ in their tissue distribution and between sexes, as well as in their responsiveness to environmental challenges. Furthermore, *timstn1* expression in the fish brain was not correlated to its expression in the muscles, suggesting that the regulation of *mstn* genes expression in Nile tilapia is gene- and tissue-specific. As various environmental challenges affected the expression of both *timstn* genes, we suggest that the MSTN system is involved in the regulation of Nile tilapia response to external conditions.

## Data Availability Statement

The datasets generated for this study can be found in the GenBank MN708486.

## Ethics Statement

The animal study was reviewed and approved by Agricultural Research Organization (ARO) Committee for Ethics in Using Experimental Animals, Approval number: 775/18 IL.

## Author Contributions

JB designed research. AS-H, GA, KT, and TN performed research. JB, AS-H, and GA analyzed data. JB and AS-H wrote the paper.

### Conflict of Interest

The authors declare that the research was conducted in the absence of any commercial or financial relationships that could be construed as a potential conflict of interest.
